# Aging reduces glial uptake and promotes extracellular accumulation of Aβ from a lentiviral vector

**DOI:** 10.3389/fnagi.2014.00210

**Published:** 2014-08-15

**Authors:** Wenjuan Zhao, Jiguo Zhang, Elizabeth G. Davis, G. William Rebeck

**Affiliations:** ^1^School of Pharmacy, Shanghai Jiao Tong UniversityShanghai, China; ^2^Department of Pharmacology, School of Pharmacy, Taishan Medical UniversityTaian, China; ^3^Department of Neuroscience, Georgetown University Medical CenterWashington, DC, USA

**Keywords:** APOE, microglia, intraneuronal amyloid-beta, lentivirus, plaque, amyloid, knock-in

## Abstract

We used a lentiviral system for expressing secreted human Aβ in the brains of young and old APOE knock-in mice. This system allowed us to examine Aβ metabolism *in vivo*, and test the effects of both aging and APOE genotype, two of the strongest risk factors for Alzheimer’s disease. We injected the Aβ_1-42_ lentivirus into the motor cortex of young (2 month old) and old (20–22 month old) APOE3 and APOE4 mice. After 2 weeks of lentiviral expression, we analyzed the pattern of Aβ accumulation, glial activation, and phosphor-tau. In young mice, Aβ accumulated mainly within neurons with no evidence of extracellular Aβ. Significantly higher levels of intraneuronal Aβ were observed in APOE4 mice compared to APOE3 mice. In old mice, APOE4 predisposed again to higher levels of Aβ accumulation, but the Aβ was mainly in extracellular spaces. In younger mice, we also observed Aβ in microglia but not astrocytes. The numbers of microglia containing Aβ were significantly higher in APOE3 mice compared to APOE4 mice, and were significantly lower in both genetic backgrounds with aging. The astrocytes in old mice were activated to a greater extent in the brain regions where Aβ was introduced, an effect that was again increased by the presence of APOE4. Finally, phospho-tau accumulated in the region of Aβ expression, with evidence of extracellular phospho-tau increasing with aging. These data suggest that APOE4 predisposes to less microglial clearance of Aβ, leading to more intraneuronal accumulation. In older brains, decreased clearance leads to more extracellular Aβ, and more downstream consequences relating to astrocyte activation and phospho-tau accumulation. We conclude that both aging and APOE genotype affect pathways related to Aβ metabolism by microglia.

## INTRODUCTION

Aging is the most notable risk factor for AD: the incidence of AD increases exponentially with aging, particularly affecting the population after age 60 (Reitz and Mayeux, 2014). Pathologically, the AD brain is characterized by the accumulation of the Aβ peptide in amyloid plaques, the accumulation of phosphorylated tau in neurofibrillary tangles, and the loss of synapses (Spires-Jones and Hyman, 2014). Genetically, AD is most strongly affected by the APOE gene: inheritance of the common APOE-ε4 allele increases risk of AD three fold, and over 10-fold in individuals with two APOE-ε4 alleles (Lambert et al., 2013). Whether APOE and aging affect pathogenesis of AD via similar or distinct pathways is not known.

Animal models of AD have been used to investigate the effects of APOE on AD pathogenesis. Transgenic AD mouse models expressing the different APOE isoforms showed earlier accumulation of amyloid in the presence of APOE-ε4 (Kim et al., 2009), consistent with higher levels of amyloid seen in humans with APOE-ε4 (Rebeck et al., 1993; Schmechel et al., 1993; Reiman et al., 2009). These studies have led to the hypothesis that APOE genotype affects clearance of Aβ, into glia or into the periphery (Castellano et al., 2011). Cell culture experiments also show that apoE3 promotes cellular uptake of Aβ in microglia (Mandrekar et al., 2009; Lee et al., 2012). These kinds of approaches have supported a role of APOE in altering Aβ clearance.

It is difficult to examine the effects of aging on Aβ accumulation in these models of Aβ metabolism, because the animals generally express very high levels of Aβ from birth (Ashe and Zahs, 2010). We have been using a lentiviral construct for expressing Aβ at specific times and in different genetic backgrounds (Burns et al., 2009; Khandelwal et al., 2011; Lonskaya et al., 2013). This construct expresses Aβ containing a signal peptide, leading to secretion of Aβ from infected neurons (Rebeck et al., 2010). Using this construct, we found that infection of 9 month old APOE4 knock-in mice led to higher levels of intraneuronal Aβ compared to APOE3 mice (Zhao et al., 2014). Interestingly, APOE3 mice showed a greater number of microglia containing the introduced Aβ compared to the APOE4 mice (Zhao et al., 2014). Neither wild-type mice nor either APOE mouse model showed evidence of extracellular Aβ accumulation (Rebeck et al., 2010; Zhao et al., 2014). We hypothesized that apoE3 promoted clearance of Aβ by microglia, while apoE4 favored less glial clearance and more intraneuronal accumulation.

We now used this system to examine the effects of aging on Aβ metabolism. We infected young (2 month old) and old (20–22 month old) mice of APOE3 or APOE4 genotypes with this lentiviral Aβ construct, and measured pathogenic effects. The aged mice showed extracellular accumulation of Aβ, and greater downstream effects on astrocyte activation and phospho-tau levels. These differences were generally exacerbated by the presence of APOE4. We conclude that APOE4 and aging both lead to less microglial uptake of Aβ and more pathogenic effects of Aβ.

## MATERIALS AND METHODS

### ANIMALS

Female APOE3 and APOE4 mice at the age of 2 months (young) and 20–22 months (old) were used in the present study. APOE knock-in mice, created by gene targeting, were the gift of Patrick Sullivan (Sullivan et al., 1997). These mice express each of the human APOE isoforms under the control of endogenous murine APOE regulatory sequences. All animals were bred and maintained in a temperature and humidity controlled vivarium at Georgetown University Medical Center, with ad libitum access to food and water on a 12 h light dark cycle. All experiments were approved and conducted according to Georgetown University Animal Care and Use Committee. Every effort was made to minimize suffering and the number of animals used for this study.

### LENTIVIRAL GENERATION

A gene transfer animal model was generated by using lentiviral delivery Aβ_1-42_ expression to specific brain regions that results in intracellular protein accumulation (Rebeck et al., 2010; Zhao et al., 2014). The lentivirus expresses Aβ_1-42_ with a signal peptide cloned into a pLenti6-D-TOPO plasmid under the control of the cytomegalovirus promoter (Burns et al., 2009). Lentivirus was generated in HEK293FT cells, and the supernatants were collected, concentrated by centrifugation, resuspended in Hank’s Balanced Salt Solution, and aliquoted in autoclaved tubes. To assure that the same number and quality of viral particles were injected, we used the same preparation of virus and injected it into the young and old APOE3 and APOE4 mice over a period of 2 days.

### STEREOTAXIC INJECTION

As in previous studies, we performed stereotaxic injection of Aβ_1-42_ lentivirus to primary motor cortex, which generated intraneuronal expression of Aβ_1-42_ in brain cortex (Burns et al., 2009; Rebeck et al., 2010; Herman et al., 2011; Algarzae et al., 2012; Lonskaya et al., 2013; Zhao et al., 2014). Motor cortex was chosen because of the reliability of the injection site, and because, in AD brains, Aβ deposits typically occur first in neocortical areas (Braak and Braak, 1991; Thal et al., 2002; Jack et al., 2009, 2010). Our earlier studies with this lentiviral Aβ construct using a control lentiviral LacZ construct demonstrated that 2 weeks after injection, control injections showed no evidence of Aβ deposition, glial activation of phospho-tau induction (Burns et al., 2009; Rebeck et al., 2010; Herman et al., 2011).

Animals were anesthetized with intraperitoneal injection of a cocktail of ketamine and xylazine (100 and 10 mg/kg, respectively). Stereotaxic surgery was then performed to inject the lentivirus encoding Aβ_1-42_ into the left side of the primary motor cortex of young and old APOE3 or APOE4 mice, as described (Hebron et al., 2013). The stereotaxic coordinates for the primary motor cortex of mice were 1.6 mm lateral (left), 1.6 mm ventral and 0.5 mm anterior. Viral stocks were injected through a microsyringe pump controller (Micro4) using total pump (World Precision Instruments, Inc.) delivery of 6 μl at a rate of 0.2 μl/min. The needle remained in place at the injection site for an additional minute before slow removal over 2 min.

### FIXATION AND TISSUE PROCESSING

2 weeks post-injection, the mice were deeply anesthetized by intraperitoneal injection of ketamine-xylazine and then fixed by transcardial perfusion with PBS; pH 7.4), followed by 4% paraformaldehyde. Brains were removed and post-fixed in 4% paraformaldehyde at 4°C overnight, incubated in two sequential 30% sucrose solutions at 4°C for 24 h each, frozen on dry ice, and coronal sections of the brain were cut (30 μm thick) on a sliding microtome (Thermo Scientific Microm HM 430). Sections were stored in cryoprotectant (30% glycerin, 30% ethylene glycol, in 0.1 M PBS) at -20°C. A total of *n* = 24 mice (*n* = 6 mice for each group) were used for immunohistochemistry.

### ANTIBODY AND REAGENTS

Monoclonal antibody MOAB2 and biotinylated MOAB2 (mouse IgG2b, 0.5 mg/ml) were used for the determination of lentiviral Aβ_1-42_ in immunofluorescence and DAB staining, respectively, (Youmans et al., 2012). Rabbit anti-NeuN antibody (Bioss, Inc.), rabbit anti-GFAP antibody (Invitrogen, CA, USA) and rabbit anti-Iba1 antibody (Wako Pure Chemical Industries) were used for the identification of neurons, astrocytes and microglial cells, respectively. Phosphorylated tau was detected by a rabbit anti-tau [pSpS199/202] phospho-specific antibody AT8 (Invitrogen, CA, USA). Biotinylated goat anti-rabbit secondary antibodies (Vectastain, Vector Laboratories) were used for DAB immunostaining of GFAP and phosphorylated tau. Fluorescent secondary antibodies: Alexa Fluor 594 donkey anti-mouse and Alexa Fluor 488 donkey anti-rabbit were from Invitrogen (CA, USA). The antibodies were diluted in PBS containing 0.25%Triton X-100 + 2% bovine serum albumin + 0.005% sodium azide.

### DAB IMMUNOSTAINING

For DAB immunohistochemistry, 30 μm coronal brain sections from young and old APOE3 and APOE4 mice were rinsed in 0.1 M PBS (6 × 10 min), incubated in quench peroxidase (10% methanol, 3% hydrogen peroxide in 1 × PBS) for 20 min and permeabilized with PBS containing 0.25% Triton X-100. Afterward, sections were incubated with 10% normal horse serum for 1 h at room temperature to block nonspecific surfaces. Sections were then incubated with biotinylated MOAB2 (mouse, 1:1000 dilution of 0.5 mg/ml stock), specific primary antibody to GFAP (rabbit, 1:1000) or phosphorylated tau (rabbit, 1:10 000) at 4°C overnight. After incubation, samples were washed in PBS (3 × 10 min), incubated with biotinylated goat anti-rabbit secondary antibody (1:200) for GFAP and phosphorylated tau for 1 h. Sections were then washed with PBS (3 × 10 min), incubated with avidin–biotin complex (Vector Laboratories) for 1 h, washed in PBS (2 × 15 min) and rinsed in 0.1 M Tris–HCl (pH 7.5) for 3 min. After that, reaction products were visualized using 0.1 M Tris-HCl (pH 7.5) containing 0.05% DABtetrahydrochloride and 0.003% hydrogen peroxide. Sections were then washed in 0.1 M Tris-HCl (pH 7.5) buffer (3 × 5 min), mounted onto glass slides, air dried overnight, dehydrated through a series of graded alcohols, cleared in xylene and cover-slipped with permount. Bright-field images were taken on a Zeiss Axiophot microscope (Carl Zeiss).

### NISSL STAINING

Tissue sections were counter-stained with a Nissl stain (cresyl violet) after DAB immunostaining to recognize cellular details. Coverslips were gently removed with xylene, brain sections were rehydrated with decreasing ethanol concentrations (100, 100, 95, 70%) for 5 min each, washed with distilled water for 5 min and treated with 0.1% cresyl violet acetate solution for 5 min at room temperature. The sections were then dehydrated with ascending series of ethanol (70, 95, 100, 100%), treated with xylene and cover-slipped with permount. Bright-field images were taken with an Olympus BX51 microscope and DP-72 CCD camera.

### IMMUNOFLUORESCENCE AND CONFOCAL MICROSCOPY

A series of double label immunofluorescence experiments were conducted to determine the colocalization characteristics of lentiviral Aβ_1-42_ with specific cell-type antibodies. Brain sections were incubated in the mixture of two primary antibodies: MOAB2 (mouse, 1:1000) and either NeuN (rabbit, 1:1000), GFAP (rabbit, 1:1000), or Iba1 (rabbit, 1:100), overnight 4°C. Sections were then washed in PBS (6 × 10 min) and incubated with the mixture of two fluorophore-conjugated secondary antibodies of Alexa Fluor 594 donkey anti-mouse, and Alexa Fluor 488 donkey anti-rabbit at dilution of 1:1000 for 1 h at room temperature in the dark. Images were captured on a Zeiss LSM 510 confocal microscope.

### ANALYSIS OF IMMUNOSTAINING

The region for quantitative analysis was in the ipsilateral cortex nearest to the injection site of Aβ_1-42_ lentivirus, which contains most MOAB2-positive neurons. Analysis of Aβ by DAB staining occurred in two sections from each animal nearest the injection site (sections were subsequently counterstained for Nissl). The three immunofluorescent doubles stains of Aβ with individual cell-type markers were conducted in single sections from each animal, but using sections near the injection site because the Aβ assay was more sensitive. DAB immunostains for astrocytes (more proximal to the injection site) and the phospho-tau epitope (more distal to the injection site) were conducted on sections from a subset of at least three brains.

Images were captured at 40× magnification using a Zeiss LSM 510 confocal microscope across an area of 675 μm × 675 μm. As in our previous study (Zhao et al., 2014), we performed manual quantification of MOAB2-positive cells, Iba1-positive cells, and MOAB2/Iba1-double-positive cells, following a modified procedure (Cerbai et al., 2012). We took the following steps to guarantee accurate counts: (1) The region we chose for quantitative analysis was in the ipsilateral cortex nearest to the injection site of Aβ_1-42_ lentivirus, which contains most MOAB2-positive neurons. (2) The pictures of nine 40× microscopic fields (675 μm × 675 μm) were taken automatically by a two-dimension scanning mechanism microscope stage. (3) The numbers of MOAB2- positive, Iba1-positive and MOAB2-/Iba1-double-positive cells were manually counted by a blinded investigator. (4) The cell numbers were determined in three sections per animal, and the average of the counts thus obtained was recorded from young APOE3, old APOE3, young APOE4, and old APOE4 groups (*n* = 6 mice in each group).

### STATISTICAL ANALYSIS

Fisher’s exact test was performed to test the frequency difference of MOAB2 immunostaining (positive versus negative) between groups of young APOE3, old APOE3, young APOE4, and old APOE4 mice. Two-way ANOVA was used to examine differences in numbers of MOAB2-, Iba1-, and MOAB2-/Iba1-double immunopositive cells among groups, followed by Tukey’s *post hoc* analyses. Data were expressed as mean ± SEM unless otherwise specified. The statistical significance criterion of *P*-value was 0.05.

## RESULTS

### Aβ IN GLIA IN YOUNG MICE AND ITS EXTRACELLULAR ACCUMULATION IN AGED MICE

Intraneuronal accumulation of Aβ is a potentially important factor participating in AD pathogenesis (LaFerla et al., 2007; Bayer and Wirths, 2010; Wirths and Bayer, 2012). Our previous work demonstrated that intracellular accumulation of human Aβ_1-42_ was enhanced in the cerebral cortex of eight to 9-month old APOE4 mice when compared to APOE3 mice 2 weeks after injection of an Aβ_1-42_ lentivirus (Zhao et al., 2014). In the current study, Aβ_1-42_ lentivirus was injected into the primary motor cortex of young (2 month old) APOE3 and APOE4 mice, as well as old (20–22 month old) APOE3 and APOE4 mice. Aβ was measured 2 weeks after the injection.

**FIGURE 1:**
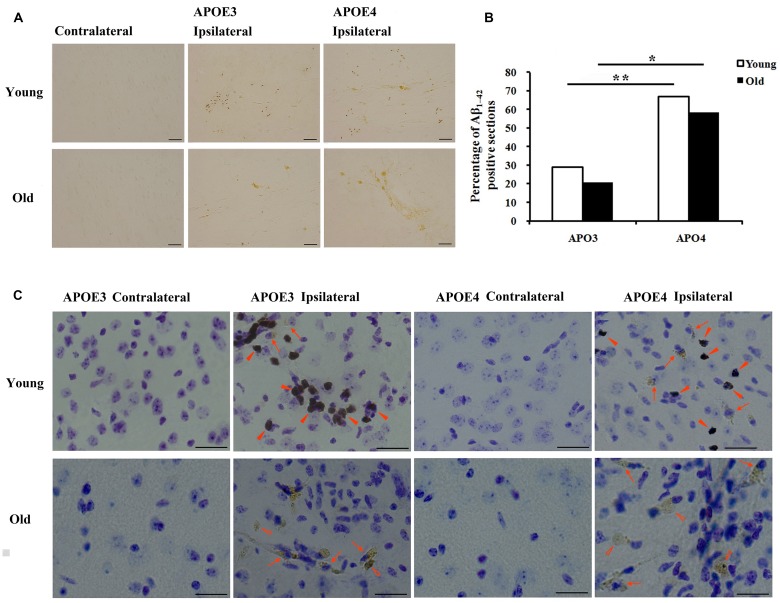
**Age and APOE genotype affect accumulation of Aβ_1-42_ after lentivirus injection. (A)** Representative images of DAB staining (brown) for biotinylated anti-Aβ antibody, MOAB2 on the contralateral and ipsilateral (left) brain cortex near the injection area from young (2 month old) APOE3, young APOE4, old (20–22 months old) APOE3, and old APOE4 mice. Scale bars: 50 μm. **(B)** The percentage of MOAB2-positive sections was significantly higher in APOE4 mice than that in APOE3 mice (**P < 0.01, young APOE4 *versus* young APOE3; *P < 0.01, old APOE4 versus old APOE3, Fisher’s exact test). **(C)** Combination of DAB-labeling immunocytochemistry for MOAB2 (brown) with Nissl staining (blue) in the contralateral and ipsilateral cortex near the injection site of Aβ_1-42_ lentivirus on the brain sections. Scale bars: 50 μm. Arrows and indicate intracellular MOAB-2 immunopositive staining in neuron-like cells; solid arrowheads indicate intracellular MOAB-2 immunopositive staining in glia-like cells in the ipsilateral cortex of young APOE3- and APOE4 mice; hollow arrowheads indicate extracellular MOAB-2 immunopositive staining in the ipsilateral cortex of old APOE3- and APOE4 mice.

Immunohistological staining with MOAB2, a pan-specific antibody to Aβ_42_ (Youmans et al., 2012) showed that the level of Aβ was strong in the ipsilateral cortex near the injection area and absent in the contralateral cortex (**Figure 1A**). Seven of 24 (29%) sections from young APOE3 mice, 16 of 24 (67%) from young APOE4 mice, 5 of 24 (21%) from old APOE3 mice, and 14 of 24 (58%) from old APOE4 mice were positively stained for MOAB2. Consistent with our previous results from APOE3 and APOE4 mice (Zhao et al., 2014), we found there were significantly more MOAB2-positive sections in APOE4 mice than in APOE3 mice, in both the young and old cohorts (**Figure 1B**, ^∗^*P* < 0.05, old APOE4 versus old APOE3; ^∗∗^*P* < 0.01, young APOE4 versus young APOE3; Fisher’s exact test). There were no significant differences between young and old groups (**Figure 1B**). Sections stained with DAB for Aβ (brown) were also counterstained with Nissl (blue; **Figure 1C**). In addition to punctate accumulation in neuron-like cells (arrows), Aβ was also strongly present in glia-like cells (solid arrowheads) in young APOE3 and APOE4 mice. Conversely, in old APOE3 and APOE4 mice, Aβ was observed extracellularly (hollow arrowheads), with less evidence of glial Aβ (**Figure 1C**). These deposits were more of the size and shape of individual cells and did not appear like spherical amyloid plaques.

### AGE-RELATED CHANGES OF INTRACELLULAR Aβ ACCUMULATION IN NEURONS AND MICROGLIA

**FIGURE 2:**
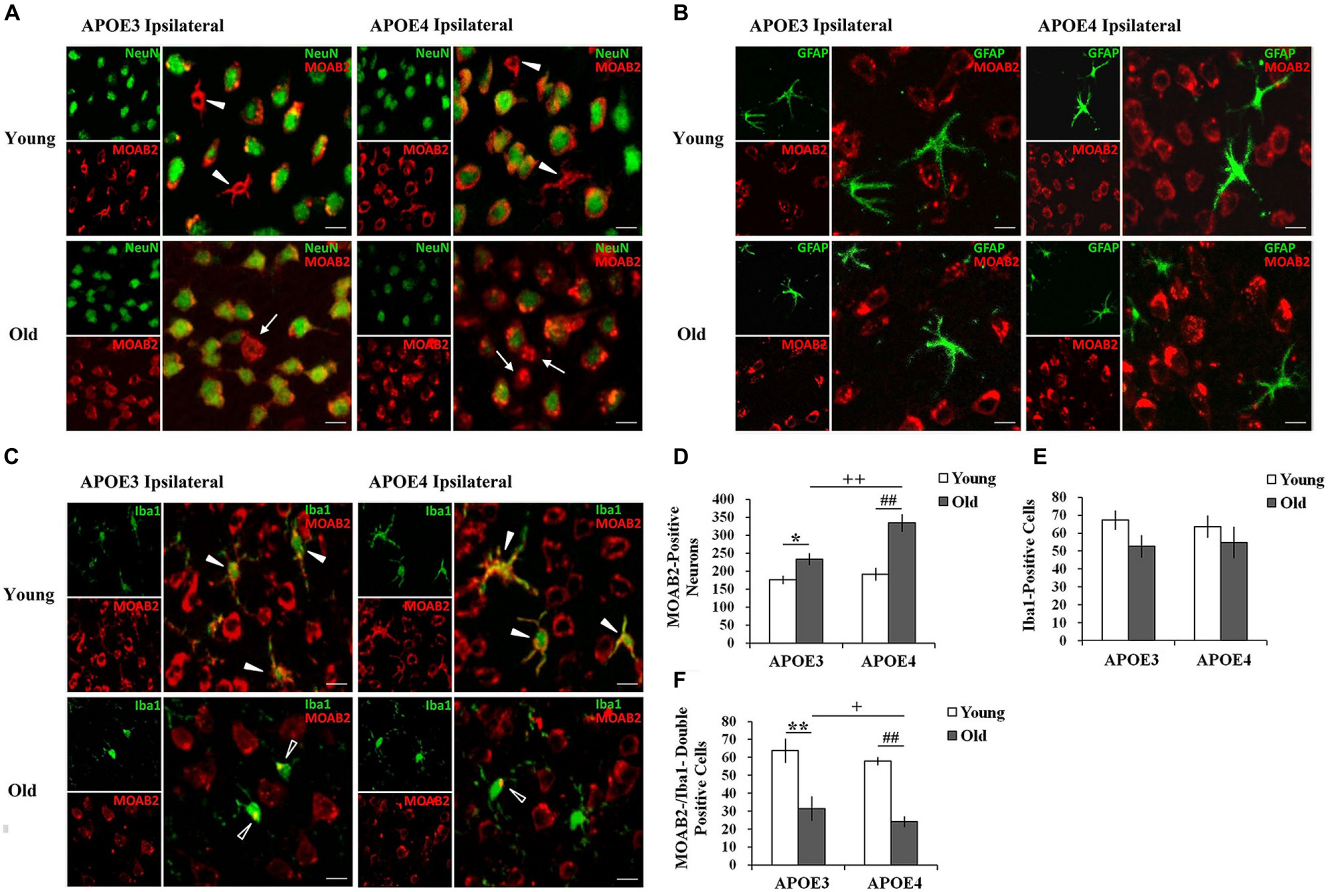
**Age and APOE genotype affect Aβ_1-42_ accumulation in neurons and glia. (A)** Aβ_1-42_ (MOAB2, red) is mainly present in cortical neurons (NeuN, green) of all mice 2 weeks after injection of lentivirus. Solid arrowheads indicate intracellular MOAB-2 immunopositive staining in glia-like cells in the ipsilateral cortex of young APOE3 and APOE4 mice; arrow indicate extracellular MOAB-2 immunopositive staining in the ipsilateral cortex of APOE3 and APOE4 mice. Scale bar: 10 μm. **(B)** Aβ_1-42_ (MOAB2, red) was not observed in astrocytes (GFAP, green) of any mice. Scale bar: 10 μm. **(C)** Aβ_1-42_ (MOAB2, red) was present in some microglia (Iba1, green) of all mice. Solid arrowheads indicate intracellular MOAB-2 immunopositive staining widespread throughout the somata and branched processes of microglia in the ipsilateral cortex of young APOE3 and APOE4 mice; hollow arrowheads indicate MOAB-2 immunopositive staining mainly in the microglia somata in the ipsilateral cortex of old APOE3 and APOE4 mice. Scale bar: 10 μm. **(D)** The average numbers of MOAB2-positive neurons per mouse in the ipsilateral cortex near the injection site of Aβ_1-42_ lentivirus. There were significantly more MOAB-2 positive neurons in APOE4 brains compared to APOE3 brains, and in older brains compared to younger brains. **(E)** N o significant differences were observed for the numbers of microglia in the ipsilateral cortex near the lentiviral injection site, as determined from Iba1 stains. **(F)** MOAB2-positive microglia (MOAB2-/Iba1-double-positive cells) per mouse in the ipsilateral cortex near the injection site. Data are expressed as mean + SEM and were analyzed by two-way ANOVAs followed by Tukey’s *post hoc* analyses. **P < 0.01, young APOE3 versus old APOE3; *P < 0.05, young APOE3 versus old APOE3; ^##^*P* < 0.01, young APOE4 versus old APOE4; ^++^*P* < 0.01, old APOE3 versus old APOE4; ^+^*P* < 0.05, old APOE3 versus old APOE4.

3,3-diaminobenzidine immunostaining only shows the areas with high levels of Aβ, so we moved to immunofluorescence to demonstrate more of the Aβ in the tissue. Using immunohistochemical markers, we examined which cells accumulated Aβ in both the young and the old mouse brains. Double immunostaining for Aβ_1-42_ (MOAB2, red) and markers of different cell-types (NeuN/ Iba1/ GFAP, green) showed intracellular accumulation of Aβ in ipsilateral cortical neurons (NeuN, **Figure 2A**) and in microglia (Iba1, **Figure 2C**) near the injection site, but not in astrocytes (GFAP, **Figure 2B**) of all mice. Although the immunopositive staining for Aβ_1-42_ (MOAB2, red) was mostly found in neurons of both young and old mice with a perinuclear localization (**Figure 2A**), a small amount was also present in glia-like cells in young mice (solid arrowheads). This glial accumulation of Aβ was further evidenced by double immunostaining of MOAB2 and Iba1 (**Figure 2C**). In the young APOE3 and APOE4 mice, the Aβ was widespread throughout the somata and branched processes of the microglia (solid arrowheads, **Figure 2C**). Conversely, in aged mice, Aβ localized mainly in the microglia somata but not in the processes (hollow arrowheads, **Figure 2C**). Importantly, Aβ was also localized extracellularly in old APOE3 and old APOE4 mice (arrows, **Figure 2A**), indicating the extracellular accumulation of Aβ only in aged mice.

From these double stained images, we were able to quantify numbers of cells with evidence of intracellular Aβ. The number of MOAB2-positive neurons was significantly higher in old APOE4 mice compared to old APOE3 mice (^++^*P* < 0.01, old APOE3 versus old APOE4, two-way ANOVAs followed by Tukey’s *post hoc* analyses, **Figure 2D**). We did not observe any significant difference in the numbers of MOAB2-positive neurons between young APOE3 and young APOE4 mice (**Figure 2D**). When comparing young to old mice, we observed significantly higher numbers of MOAB2-positive neurons in old APOE3 and old APOE4 mice compared to their young counterparts (**Figure 2D**). The numbers of MOAB2-positive neurons were significantly less in the young APOE3 (176 ± 10) and young APOE4 (192 ± 15) mice compared to old APOE3 (233 ± 16) and old APOE4 (334 ± 22) mice (^∗^*P* < 0.05, young APOE3 versus old APOE3; ^##^*P* < 0.01, young APOE4 old APOE4, two-way ANOVAs followed by Tukey’s *post hoc* analyses, **Figure 2D**). Thus, aging contributed to greater accumulation of Aβ in neurons, and inheritance of APOE4 exacerbated that effect in the old mice.

By Iba1 analyses, we did not observe any difference in the total number of microglia depending on APOE genotype or age (**Figure 2E**). However, the number of MOAB2/Iba1-positive microglia in the old APOE4 mice was significantly less than in old APOE3 mice (^+^*P* < 0.05, two-way ANOVAs followed by Tukey’s *post hoc* analyses, **Figure 2F**). We did not observe any difference in the numbers of MOAB2/Iba1-positive microglia between young APOE3 and young APOE4 mice (**Figure 2F**). There were fewer Aβ containing microglia in old APOE3 and old APOE4 mice compared to young mice (**Figure 2F**). The numbers of MOAB2/Iba1-positive microglia in the young APOE3 mice (64 ± 6) and young APOE4 mice (58 ± 7) were significantly higher than in the old APOE3 mice (32 ± 2) and old APOE4 mice (24 ± 3; ^∗∗^*P* < 0.01, young APOE3 versus old APOE3; ^##^*P* < 0.01, young APOE4 old APOE4, two-way ANOVAs followed by Tukey’s *post hoc* analyses, **Figure 2F**). These results suggested that aging reduced uptake of Aβ by microglia and promoted intraneuronal accumulation of Aβ, an effect again increased in APOE4 mice.

### AGING PROMOTED ACTIVATION OF CORTICAL ASTROCYTES SURROUNDING Aβ

**FIGURE 3:**
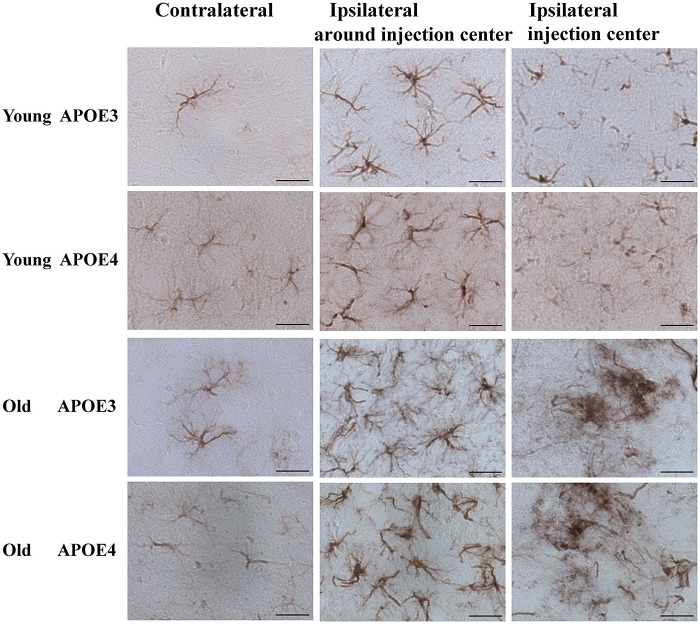
**Age and APOE genotype affect induction of astrocytic activation by Aβ_1-42_.** Representative DAB staining images of GFAP for astrocytes in the contralateral cortex, ipsilateral cortex near the injection site and injection center injection center and cortex near the injection site of Aβ_1-42_ lentivirus on brain sections of young APOE3, young APOE4, old APOE3, and old APOE4 mice 2 weeks after injection. Scale bar: 50 μm.

Although Aβ_1-42_ immunoreactivity was not observed in astrocytes (**Figure 2**), we monitored the activation of cortical astrocytes in young and aged APOE3 and APOE4 mice. Immunohistochemical staining of GFAP revealed the morphological changes of activated astrocytes associated with the aging and APOE genotype. In the control (contralateral) cortex, GFAP positive astrocytes were characterized by slim bodies and slender processes extending radially from the cell body (**Figure 3**, first column). In the ipsilateral cortex around the injection site of the Aβ_1-42_ lentivirus (**Figure 3**, second column), there was increased GFAP staining, with increased astrocyte cell body size, and dendrite processes, signs of astrocyte activation, in all animals. Compared to the young animals, activated astrocytes in the ipsilateral cortex of both APOE3 and APOE4 aged mice exhibited intense GFAP immunoreactivity and highly ramified morphology with hypertrophic processes, as well as increased astrocyte numbers. Activation of astrocytes with dense staining of the enlarged cell bodies and the highlighted cell processes was much more prominent in old APOE4 mice. In the cortex near the lentivirus injection center (**Figure 3**, third column), GFAP immunoreactive astrocytes in young APOE3 and young APOE4 mice revealed less intense labeling with indistinct cell bodies and cellular processes. In contrast, cortical astrocytes in the old APOE3 and APOE4 mice were hypertrophic, intensely GFAP immunoreactive, with swollen cell bodies.

### AGING INCREASED TAU ACCUMULATION INDUCED BY Aβ_1-42_

**FIGURE 4:**
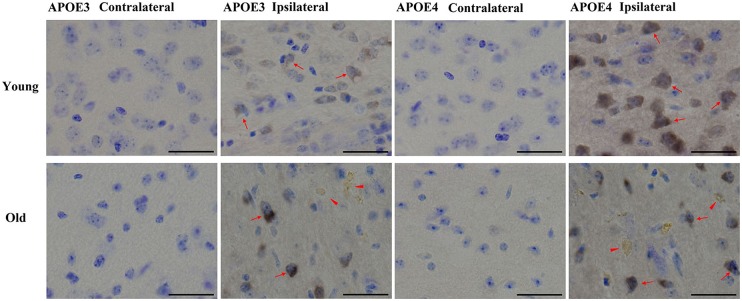
**Tau phosphorylation induced by Aβ_1-42_.** Representative images of DAB staining for phospho-tau pSer199/202 antibody (AT8, brown) with Nissl staining (blue) in the contralateral or ipsilateral cortex near the injection site of Aβ_1-42_ lentivirus on brain sections of young APOE3, young APOE4, old APOE3, and old APOE4 mice 2 weeks after injection. Red arrows indicate phosphorylated tau in neurons. Solid red arrowheads indicate extracellular phosphorylated tau in the ipsilateral cortex of old APOE3- and old APOE4 mice. Scale bars: 40 μm.

We have previously shown that AT8-positive staining is detected in the ipsilateral cortex of young mice (Zhao et al., 2014). Here we observed a similar pattern of phosphorylated tau induced by lentiviral Aβ_1-42_ in the ipsilateral cortex, but not contralateral cortex, in young (2 month old) and old (20–22 month old) APOE3 and APOE4 brains (**Figure 4**). Young APOE3 mice demonstrated slight, but clearly observable AT8-positive tau immunoreactivity in cortical neurons near the injection site (arrows). The staining for AT8 in the young APOE4 mice was also clearly visible in the cytoplasm of many neurons (upper panels, **Figure 4**). The intracellular staining of hyperphosphorylated tau was not evenly distributed: some regions were heavily labeled with intracellular aggregate-like structures, but adjacent regions showed little staining both in young and old mice. The older brains also demonstrated extracellular AT8-positive tau deposits (arrowheads), as well as strong intracellular distributions (arrows, **Figure 4**).

## DISCUSSION

This lentiviral system for the controlled expression of secreted Aβ into different tissues has allowed us to test how Aβ is metabolized in the brains of old versus young mice, and whether the metabolism was affected by APOE genotype. Consistent with what we previously found (Zhao et al., 2014), APOE4 brains accumulated more Aβ compared to APOE3 brains. Importantly, aging caused a change in the accumulation of Aβ: Aβ immunoreactivity in older brains showed more evidence of extracellular deposits, while the younger brains showed more evidence of intracellular Aβ. Aged brains also showed many more neurons with measureable Aβ compared to younger brains. These data suggest that an aged brain is less efficient at clearance of intraneuronal Aβ, and more prone to the accumulation of extracellular Aβ.

We investigated the tissues further to test whether glial accumulation of Aβ differed in aged brains, since glial clearance of Aβ is a mechanism that may be important for preventing Aβ accumulation in aged brains (Guenette, 2003; Mandrekar et al., 2009; Lai and McLaurin, 2012; Thal, 2012). We found that the brains of the younger mice had significantly higher levels of Aβ in microglia, evidence of better clearance through this mechanism. Indeed, numbers of microglia containing Aβ were reduced in aged brains. We did not observe Aβ in astrocytes, suggesting that astrocytic clearance is less important *in vivo*, or that astrocytes metabolize Aβ more efficiently than microglia. Since lentiviral Aβ expression is primarily and perhaps exclusively neuronal, we propose the large amount of microglial Aβ results from the combination of eﬄux of Aβ from neurons [perhaps through mechanisms relating to the cholesterol eﬄux molecule, ABC-A1 (Koldamova et al., 2014)], and then the subsequent uptake by microglia.

The role of APOE in this clearance mechanism is demonstrated in this study and our previous work (Zhao et al., 2014). We found that the neuronal accumulation of Aβ_1-42_ is highest in APOE4 mice, supporting the hypothesis that apoE is involved in clearance of Aβ, and apoE4 is less efficient at this process (Basak et al., 2012). Our measures of Aβ in microglia suggest that the balance of neuronal and microglial metabolism of Aβ is altered in APOE4 mice, lowering microglial clearance and increasing intraneuronal accumulation. We hypothesize that astrocytic apoE associates with neurons and promotes Aβ eﬄux, leading to microglial degradation of Aβ (Jiang et al., 2008; Mandrekar-Colucci et al., 2012), and that apoE4 is impaired in its ability to promote this eﬄux and clearance. The complex interplay of cell-types (astrocytic apoE aiding eﬄux of neuronal Aβ to promote microglial clearance) emphasizes the importance of addressing this model *in vivo*.

The activation of microglia can serve in a neuroprotective manner by responding and clearing invading pathogens and local debris (Glezer et al., 2007; Lehnardt, 2010; Rodgers and Miller, 2012). However, microglia become dysfunctional as a function of age and display diminished capacity for normal functions related to migration, clearance, and the ability to shift from a pro-inflammatory to an anti-inflammatory state (Vaughan and Peters, 1974; Samorajski, 1976; Sheng et al., 1998; Ye and Johnson, 1999; Sierra et al., 2007; Streit et al., 2009; Schuitemaker et al., 2012). This change in microglia potentially contributes to increased susceptibility and neurodegeneration as a function of age (Harry, 2013). Diminished microglial motility and reduced speed of an acute response to injury may be the result of cell senescence (Damani et al., 2011). Signaling between CX3CL1 and its receptor CX3CR1 is critical for microglia migration in the adult brain, and in the aged brain, levels of each are diminished (Wynne et al., 2010; Bachstetter et al., 2011). Levels of CD200 are decreased (Frank et al., 2006) suggesting of loss of the regulatory ability of neurons to maintain microglia in a quiescent state. Microglia isolated from aged mice compared to young mice also showed a reduced ability in CD47-related phagocytosis Aβ fibrils (Floden and Combs, 2011). In APOE transgenic mice, an age-related and genotype-related deficit was observed in the ability of the animals to mount a host-resistance response to hippocampal injury (Harry et al., 2000). No differences were observed between mice expressing various forms of human apoE gene at 2 months of age, but by 8 months of age, the APOE4 mice showed a reduced ability to mount a host-response and pro-inflammatory cytokine response (Harry et al., 2000). These studies demonstrate important changes with aging that could contribute to the reduced ability to clear Aβ, as evidenced in our data. We propose that these changes to Aβ clearance with aging are distinct from the processes impaired with APOE4, since reductions were observed with aging in both APOE3 and APOE4 mice.

Astrocyte activation contributes to the failure of Aβ clearance and the neurotoxic events downstream of Aβ in models of AD (Garwood et al., 2011; Yan et al., 2013). As we previously found with this system (Zhao et al., 2014), Aβ was not observed in astrocytes. The GFAP immunoreactivity showed that astrocyte morphology was affected by the presence of Aβ in an aging-dependent fashion, with APOE genotype contributing to the effects in the aged mice. Astrocytes may be responding to the accumulation of Aβ without being involved in its clearance, or astrocytes may metabolize the Aβ efficiently and thus we are unable to observe its intracellular accumulation.

Intraneuronal accumulations of Aβ may be precursors to later accumulation of amyloid plaques (Gouras et al., 2012). There may be an important role for autophagy in degrading intraneuronal Aβ (Burns et al., 2009; Khandelwal et al., 2011; Lonskaya et al., 2013), and thus limiting its conversion to extracellular deposits (Nilsson et al., 2013). Autophagic processes could be induced by several mechanisms, but the involvement of the ubiquitin ligase complex including Parkin may be particularly important (Burns et al., 2009; Khandelwal et al., 2011; Lonskaya et al., 2013). Our experiments here demonstrate that the processes that limit the accumulation of extracellular Aβ decay with aging, as no extracellular Aβ was observed at 2 months or 9 months of age (Zhao et al., 2014), but was observed after 20 months of age.

Glial activation in AD brain has been implicated in both beneficial and harmful processes (Keene et al., 2011; Aguzzi et al., 2013). We did not observe large changes in microglial activation over the 2 weeks of Aβ expression, although we have consistently observed microglial accumulation of Aβ; longer time points after lentiviral Aβ injection may be needed to observe effects on microglial activation. Changes to microglia with aging have been observed (Mosher and Wyss-Coray, 2014), and may be responsible for the changes to the accumulation of Aβ with aging in this model. We did not observe Aβ in astrocytes in these brains, but we did observe a strong change in the morphology of astrocytes in the presence of Aβ. We believe these changes reflect a very early response of astrocytes to the presence of extracellular Aβ.

Our approach has also allowed us to determine the early downstream effects of the presence of human Aβ on neuronal health. We analyzed the accumulation of the AT8 phospho-tau epitope within 2 weeks of the introduction of human Aβ. We found that in that short time, phospho-tau was induced, and that induction was stronger in the APOE4 mice compared to the APOE3 mice. Furthermore, aging led to a strong induction of AT8 positivity. These data demonstrate the power of lentiviral Aβ to recapitulate many of the hallmarks of AD: intracellular and extracellular accumulation of Aβ, microglial clearance of Aβ, astrocyte activation, and aberrant phosphorylation of tau.

Lentiviral expression constructs are allowing new ways to quickly study various proteins associated with neurodegeneration *in vivo*. These proteins include Aβ42 (Burns et al., 2009; Rebeck et al., 2010), Parkin (Algarzae et al., 2012; Hebron et al., 2013), tau (Khandelwal et al., 2012), α-synuclein (Moussa et al., 2008), and TDP-43 (Herman et al., 2012; Hebron et al., 2013). Experiments with the Aβ lentivirus have demonstrated that Parkin alters metabolism of Aβ and reduces associated neurotoxic effects by promoting autophagy pathways (Burns et al., 2009; Khandelwal et al., 2011; Algarzae et al., 2012). With lentiviral expression, the interactions of these pathogenic proteins can be quickly addressed without having to generate new transgenic mouse models. Furthermore, this system examines the metabolism of Aβ, as opposed to other transgenic systems that are affected by both Aβ production and clearance.

Our work here shows a pathway of Aβ clearance *in vivo*, with the conversion of intracellular Aβ from neurons to microglia. This pathway is impaired in animals expressing APOE4 compared to APOE3, and is degraded in aged animals compared to young animals. These data demonstrate that two of the strongest factors predisposing to AD, aging and APOE4, both have effects on the earliest stages of Aβ metabolism *in vivo*.

## AUTHOR CONTRIBUTIONS

Wenjuan Zhao infected the animals, collected brain tissue, performed immunohistochemical assays, and analyzed the images. Elizabeth Davis performed double immunofluorescence staining and Jiguo Zhang quantified immunofluorescence staining. G. William Rebeck designed the overall direction, and both Wenjuan Zhao and G. William Rebeck planning analyses and interpreting results.

## Conflict of Interest Statement

The authors declare that the research was conducted in the absence of any commercial or financial relationships that could be construed as a potential conflict of interest.

## ABBREVIATIONS

AD: Alzheimer’s disease
Aβ: Amyloid β
apoE: Apolipoprotein E
DAB, 3: 3-diaminobenzidine
PBS: phosphate-buffered saline.
